# Three Millennia of Southwestern North American Dustiness and Future Implications

**DOI:** 10.1371/journal.pone.0149573

**Published:** 2016-02-17

**Authors:** Cody C. Routson, Jonathan T. Overpeck, Connie A. Woodhouse, William F. Kenney

**Affiliations:** 1 School of Earth Sciences and Environmental Sustainability, Northern Arizona University, 625 S. Knoles Drive, PO Box 4099, Flagstaff, AZ 86011, United States of America; 2 Institute of the Environment, University of Arizona, 1064 E Lowell St., Tucson, AZ 85721, United States of America; 3 Department of Geosciences, University of Arizona, 1040 E. 4th St., Gould-Simpson, Room 208, Tucson, AZ 85721, United States of America; 4 Department of Atmospheric Sciences, University of Arizona, 1118 E. 4th St., Tucson, AZ 85721, United States of America; 5 School of Geography and Development, University of Arizona, 1064 E. Lowell St., Tucson, AZ 85721, United States of America; 6 Laboratory of Tree-Ring Research, University of Arizona, 1215 E. Lowell St., Tucson, AZ 85721, United States of America; 7 Land Use and Environmental Change Institute, University of Florida, 241 Williamson Hall, Gainesville, FL 32611, United States of America; Institute of Tibetan Plateau Research, CHINA

## Abstract

Two sediment records of dust deposition from Fish Lake, in southern Colorado, offer a new perspective on southwest United States (Southwest) aridity and dustiness over the last ~3000 years. Micro scanning X-ray fluorescence and grain size analysis provide separate measures of wind-deposited dust in the lake sediment. Together these new records confirm anomalous dustiness in the 19^th^ and 20^th^ centuries, associated with recent land disturbance, drought, and livestock grazing. Before significant anthropogenic influences, changes in drought frequency and aridity also generated atmospheric dust loading. Medieval times were associated with high levels of dustiness, coincident with widespread aridity. These records indicate the Southwest is naturally prone to dustiness. As global and regional temperatures rise and the Southwest shifts toward a more arid landscape, the Southwest will likely become dustier, driving negative impacts on snowpack and water availability, as well as human health.

## Introduction

Windblown dust has important implications for air quality, human health, and water resources [1–3]. Dust transports major and trace elements [4–6], which impact nutrient balance, soil development, terrestrial and aquatic ecosystems, and water quality [7–9]. Recent dust on snow events in the Rocky Mountains, the headwaters to major river systems that support over 60 million people [10], have decreased snow albedo, which in turn accelerates melt, decreases runoff, and reduces snow-cover duration by more than a month [3,11–12]. Dust emissions are linked with soil stability and wind strength in dust source regions [13–15]. Desert dust emissions have roughly doubled in many regions of the world during the 20^th^ century [16]. Consistent with this trend, Southwestern dustiness increased substantially with historical and modern land use [17], but recent drought has also enhanced windblown dust off undisturbed landscapes [15]. Across the Southwest, increases in airborne dust prompted extensive research on the fate and transport of dust and implications for air quality, human health, and water resources (e.g. [1–3,11–12,15,18–20]). However, these studies have either focused on a relatively short period of record or are too low-resolution to fully assess potential links between past Southwestern drought and dust mobilization. It is unclear the extent to which Southwestern dustiness is a modern phenomenon associated with human land use [17,21–23], or if comparably dusty conditions have occurred intermittently over longer timescales as suggested by regional eolian sediment features (e.g. [24]).

Dune and loess deposits indicate that some locations in the western U.S. experienced arid and dusty intervals during the Holocene [25–26]. At the same time, Southwestern tree-ring records provide strong evidence for multi-decadal-length droughts during Roman (1–400 AD) and medieval times (900–1400 AD) [27–28]. Were these past multidecadal-length droughts severe enough to mobilize dust? Some dune deposits in the Southwest may have activated in response to severe Roman and medieval droughts [24]. Evidence from the Wind River Range, Wyoming, suggests dust may have increased somewhat during medieval times [21]. However, other existing dust records with low temporal resolution in the San Juan Mountains and the Wasatch Mountains show no change in dust accumulation rates before the mid 1800s AD [17,22–23], suggesting biologic crusts in dust source areas may have been sufficient to stabilize soils [29]. Here we present a set of high-resolution records from Fish Lake in the south San Juan Mountains, Colorado to characterize the natural variability of dustiness better and to gain a better understanding of the past relationships between dust and drought in the Southwest.

## Methods

### Site description

**Fig 1:**
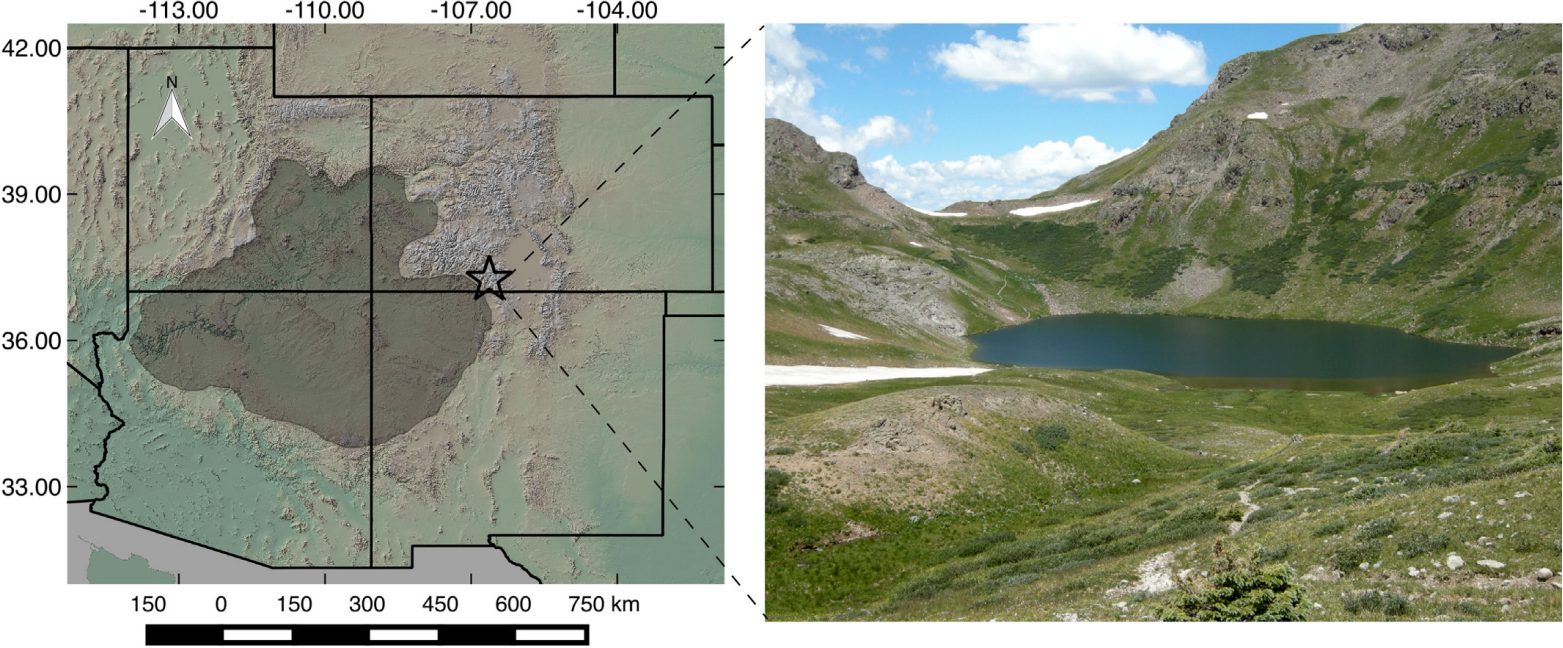
Study site location and photograph. The map shows Fish Lake (star) located in the South San Juan Mountains, Colorado, and the Colorado Plateau dominant dust source region highlighted in grey. The base map digital elevation model was obtained through the open source USGS National Map Viewer: http://viewer.nationalmap.gov/viewer/. The photograph of Fish Lake was taken in July 2009, looking to the North West down the small inlet stream. The outlet channel cuts through the rock bank on the left side of the photograph. Photograph credit Cody Routson.

Fish Lake (Fig 1; 37.25°N, 106.68°W; 3718 m elevation) is located above the treeline where prevailing southwesterly winds deposit dust from the high desert Colorado Plateau. Dust is currently deposited in the San Juan Mountains at a rate of 5–10 g m^-2^yr^-1^ [5]. The surface area of Fish Lake is 0.048 km^2^, and the total lake catchment area is 0.7 km^2^. Steep slopes on the North and East and more gentle slopes to the South and West border the lake. Rock outcrops, grass and alpine willow surround Fish Lake (Fig 1 and S1 Fig), which is located in the San Juan Volcanic Field, Conejos Formation, spanning both vent and volcanoclastic facies [30]. Vent facies on the north and eastern sides of Fish Lake are mostly flows and breccias of andesite and rhyodacite. To the south and west sides of Fish Lake, the volcaniclastic facies consist of breccias containing clasts of andesite and rhyodacite. Both volcanic geologic units have geochemistry distinct from the weathered sedimentary desert soils.

### Core sampling

In summers of 2009 and 2011 a 170 cm long core and a 30 cm long core were taken respectively from Fish Lake using Alpacka rafts and a universal gravity corer (S1 Fig). Because Fish Lake is a public resource with free access at all times, specific permission to collect sediment cores was not required. Cores were taken near the center of the lake in the deepest portion at 18.5 m deep. The sediment-water interface was preserved on the short core by siphoning off water above the sediment surface, carefully packing with a sponge to absorb water and prevent slumping. A small portion of sediment was lost from the surface of the longer core. Thus the short core was used for ^210^Pb dating (discussed below) and to extend the record to the present. The study did not involve endangered species.

### Dust and bedrock sampling

Dust samples were collected in April 2012 by sampling the snow surface and dust-rich layers in the snowpack ~1 km south of Wolf Creek Pass (On the continental divide ~27 km north-northwest from Fish Lake). Wolf Creek Pass had already experienced eight dust on snow events that winter [31]. The snowpack had deflated somewhat, but there was still continuous snow cover. The dust in snow was sampled into 1-liter acid-washed Nalgene bottles. Returning to the lab, the snowmelt was evaporated from the bottles in a drying oven at 50°C. The dried dust samples were homogenized in a mortar and pestle and compressed into pellets for micro scanning X-ray fluorescence (μXRF) analysis.

To constrain local bedrock elemental abundance, seven bedrock samples were collected predominantly from the south and east side of Fish Lake, spanning both geologic units, although the bedrock samples were all andesite and rhyodacite. Chunks of the bedrock (including a mixture of the weathered rind as well as un-weathered bedrock interior) were powdered and homogenized in a mortar and pestle pre-cleaned with an alcohol swab. The rock powder was compressed into pellets for analysis on the μXRF.

### Sediment description

Sediments in the core were composed of fine-to coarse grained, organic-poor material. The sediments were light brown in color with some fine-scale layers present, but the sediment was not varved and generally massive. The lake is located above modern tree line, but small shrub twigs and one spruce cone provided abundant material for radiocarbon dating. Organic concentrations determined through loss on ignition (combustion at 500°C for 12 hours) range between 1 and 14%. Thirty turbidites (distinct packages of sediment deposited instantaneously by floods or underwater landslides) were removed from the Fish Lake records (S2 Fig). The core and digitized thin-section photographs in ArcGIS were used to visually characterized trubidites: coarse intervals of sediment with a sharp bottom boundary and fining upward. Turbidite depths were confirmed as intervals of anomalously high μXRF Ca abundance and, less precisely due to resolution, as coarse grain size intervals. Sediment thin-sections mounted on microscope slides were digitized using a digital SLR camera through an Olympus microscope. The thin-section photographs were imported into ArcGIS and scaled to depth using μXRF line-scans on the sediment slabs. Turbidite depths were measured in ArcGIS and verified with measurements on the wet sediment core. Short and long cores were adjusted linearly to the same depth scale using marker layers and μXRF line scans to cross-correlate between cores.

### Age control

**Fig 2:**
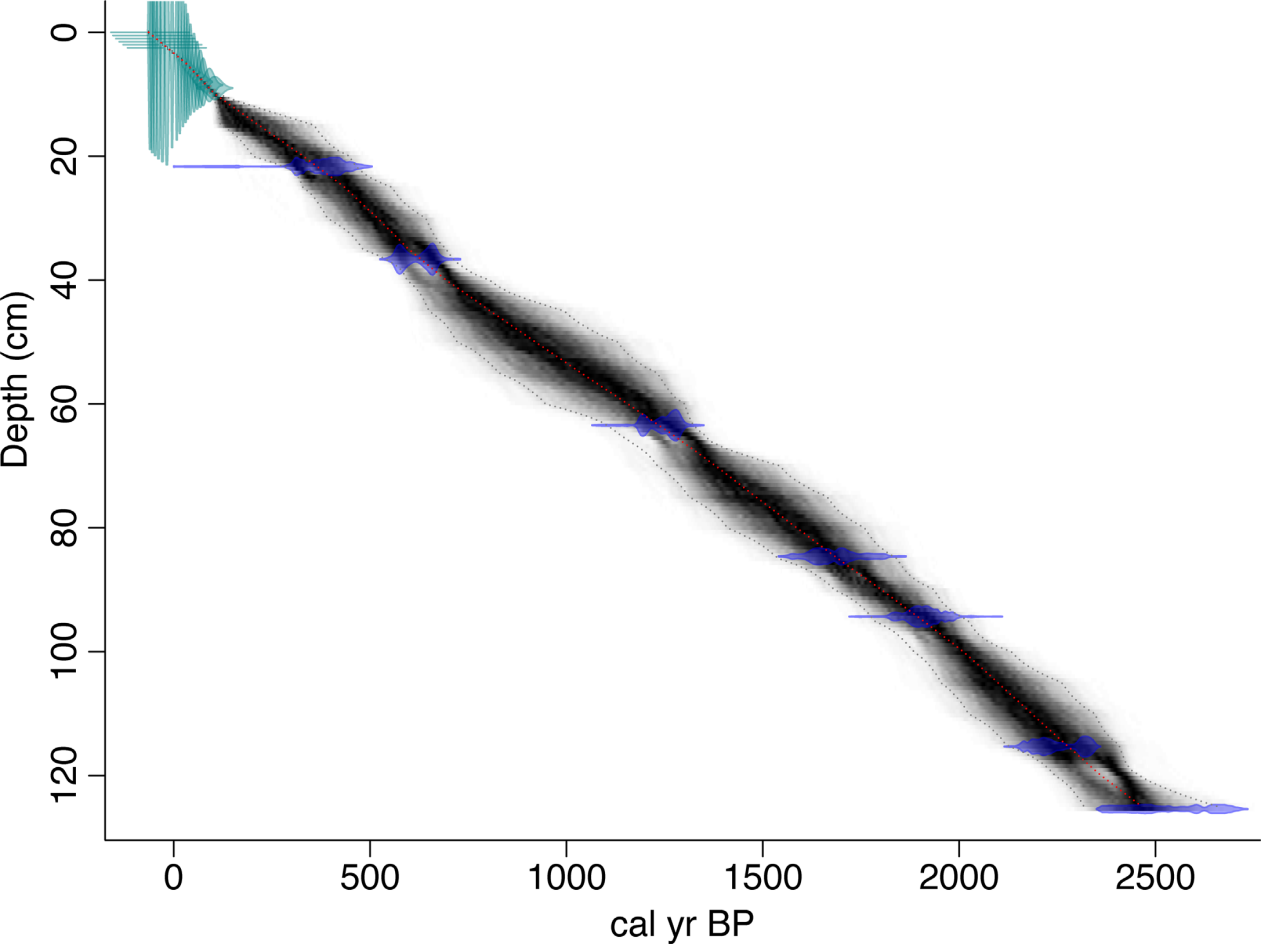
Age depth chronology. Age depth chronology based on ^210^Pb dates on the upper 9 cm (light blue), and ^14^C dates (dark blue). The red line shows the best fit of 1000 age models and 1-sigma errors are shown in the dotted grey lines. The age depth model and figure were generated using the Bayesian age-depth modeling program BACON [36].

Age control was developed using ^210^Pb and ^14^C dating. Sediments of the upper most portion of the short surface core were sampled at 0.5 cm intervals and radiometric measurements (^210^Pb and ^226^Ra) were made at the University of Florida, Land Use and Environmental Change Institute using low-background gamma counting with well-type intrinsic germanium detectors [32–33]. Sediment ages were calculated using the constant rate of supply model (S3 Fig) [34]. Age errors were propagated using first-order approximations and calculated according to Binford [35]. Radiocarbon dating on terrestrial macrofossils was used to constrain ages beyond the ^210^Pb chronology. Radiocarbon samples were combusted and analyzed at the University of Arizona’s Accelerator Mass Spectrometer facility. Marker layers were used to correlate age depths between the short and long cores. Radiocarbon ages were calibrated and age depth models were developed (Fig 2) using the Bayesian age-depth modeling R software package BACON [36]. Radiocarbon dates were calibrated using the IntCal09.14C calibration curve [37]. BACON uses the assumption that sedimentation rates are within a range characterized by a long-tailed probability distribution, and that radiocarbon ages are in stratigraphic order. BACON models sedimentation rates using a gamma autoregressive process and generates age distributions using a Markov Chain Monte Carlo Algorithm [38]. The resulting family of possible age-depth models reflect the probability associated with the analytical and calibrated radiocarbon age uncertainties.

### Grain size analysis

Particle size analysis has been widely used to characterize dust contributions to lake sediments [14,39–44]. To assess particle size distributions and help characterize the dust contribution to Fish Lake, sediments were sampled at 0.5 cm continuous intervals for grain size measurements on the short and long cores. Grain size samples were pretreated using a modification of the methods described by Dr. Donald Rodbell of Union College (http://www1.union.edu/rodbelld/grainsizeprep.htm). In a sequence of treatments, 10% HCL was used to remove potential carbonates, 30% H_2_O_2_ was used to remove organics, and 1 M NaOH was used to remove biogenic silica. Sediment samples were rinsed, centrifuged, and decanted three times between each step, following the methods used in [45]. We also added (NaPO_3_)_6_ to the samples before analysis as a dispersant to inhibit aggregation of clay-sized particles. Grain-size distributions were analyzed using a laser-diffraction Malvern Mastersizer, 2000 particle size analyzer at the University of Arizona Department of Geosciences. Each sample was measured 5 times. Dust samples were pretreated and analyzed using the same grain size protocol as the sediment. Discussed in more detail in the results section, dust particle sizes were characterized as less than or equal to 15.1μm.

### Geochemical μXRF analysis

Following on earlier work [17,39,44], μXRF was also used to characterize the contribution of dust to Fish Lake Sediment. Sediments were sampled by carefully removing wet slabs 4.5 x 2.0 x 0.5 cm in size. Acetone exchanges were used to remove water, and the slabs were imbedded in an epoxy resin. Imbedded sediment slabs were split using a diamond saw and surfaced on 600 grit sanding paper. Half of each slab was used to make glass microscope thin-sections. The other half was analyzed using an EDAX Eagle III tabletop scanning μXRF analyzer at the University of Arizona Department of Geosciences. Line scans down each slab were run using 40kv, 300μa, at 25-micron resolution, and 16 seconds of spot measurement time. Several sections did not imbed properly resulting in gaps in the μXRF records. Sub-annual μXRF measurements were averaged to 1-year resolution to help reduce instrument noise associated with the relatively short (16 second) spot measurement time. Bedrock and dust samples compressed into pellets were also analyzed using μXRF with the same instrumental settings as the sediment samples.

There were some cases of sediment shrinking when imbedding the sediment slabs. Sediment shrinking was accounted for by using the depth differences of 36 marker layers between the thin-sections and the wet core. Depths were adjusted linearly between the marker layers. Shrinkage was also visible by comparing the grain size (sampled directly from the sediment core) and the μXRF records (from the sediment slabs). Distinct marker layers such as coarse grain sizes corresponding with Ca μXRF peaks (S4 Fig), were used to check that all the records were on the same depth-scale after adjusting for the sediment shrinking.

The mineral component of Fish Lake sediment is a mixture of local terrigenous runoff, and wind deposited dust. To calculate the fraction of dust (fd) in the sediment, we applied a geochemical end-member mixing model (1) using potassium and calcium ratios in dust, local bedrock, and sediment (e.g. [44]). The μXRF dust fraction records on short and long cores were were then averaged together to extend the record to the present.

$$fd=\frac{\frac{K}{Ca}sed-\frac{K}{Ca}rock}{\frac{K}{Ca}dust-\frac{K}{Ca}rock}$$ (1)

To conceptualize the mixing model (discussed in more detail in the results section), a mean μXRF count adjustment was applied to the sediments. When analyzing the sediment, X-rays travel through epoxy imbedding resin and organic sediment in addition to the mineral component of the sediment. The epoxy resin and organic matter reduce the μXRF signal relative to the pure dust and bedrock samples (S5 Fig). Different elements are influenced slightly differently. A constant value of 40 was added to the potassium and calcium mean counts, and the value 20 was added to titanium mean counts (S5 Fig). The adjustment has no influence on the final dust record because potassium and calcium ratios were used.

### Composite record

In addition to the individual grain size and μXRF dust records, a method adapted from tree-ring techniques was applied to reduce method- and core-dependent variability. Given the reasonable coherence between the grain size and μXRF dust estimations (e.g. S6 Fig), the four records (grain size and μXRF applied to the short and long cores) were normalized by their mean and variance. Grain size dust records were then interpolated to 5-year sample resolution and the μXRF dust records were binned to 5-year sample resolution, and then all normalized records were averaged into a single site level dust reconstruction. All records were weighted equally, although the grain-size record is arguably less robust than the μXRF record. The composite record units indices, similar to a tree-ring index values resulting from the average normalized records. With sufficient instrumental dust deposition data, the dust index could be scaled linearly to deposition rate, but for now the record simply shows relative changes in dust deposition.

### Error analysis

Uncertainties in the grain-size and geochemical dust records were estimated using an ensemble approach. Analytical grain-size uncertainty was estimated by measuring each grain-size sample 5 times. A distribution of 1000 records were generated, randomly sampling from the 5 sample measurements at each sample interval down core, from which the upper and lower 95^th^ percentiles were obtained. Although this gives us a starting point for estimating error, it is challenging to assess the true magnitude of error associated with the grain size records. Grain size uncertainties are discussed in more detail in the results section below. Uncertainty in the geochemical dust records was estimated by generating 1000 estimations of dustiness for each depth by randomly sampling from the individual μXRF end-member measurements. Finally, age uncertainty for the records was estimated and integrated into the records’ uncertainty bars (e.g. [46]) using the Bayesian derived family of age models from BACON [36].

## Results

### Age control

Radiometric ^210^Pb dating provides well-constrained age control on the upper ~9 cm of surface sediments, while lower resolution ^14^C constrains ages on the remainder of the core (Table 1). The Bayesian derived age-depth model suggests only slight variability in sediment accumulation rates (Fig 2). However, decadal-scale variability in sedimentation rates would be impossible to detect given that radiocarbon age control is greater than 30 years.

**Table 1:** Radiometric dates on the Fish Lake sediment core. Radiometric dates include ^210^Pb on the upper 9 cm of the short sediment core, and radiocarbon dates on the longer sediment core.

labID	age (yr BP)	error (yr)	depth (cm)	Material	Date Type
Surface	-60	1.00	0	bulk sediment	210PB
FL0.5	-54.8	1.14	0.5	bulk sediment	210PB
FL1.0	-47.4	1.26	1	bulk sediment	210PB
FL1.5	-38.9	1.19	1.5	bulk sediment	210PB
FL2.0	-28.9	1.30	2	bulk sediment	210PB
FL2.5	-17.6	1.27	2.5	bulk sediment	210PB
FL3.0	-4.2	1.56	3	bulk sediment	210PB
FL3.5	7.7	1.87	3.5	bulk sediment	210PB
FL4.0	15.4	2.18	4	bulk sediment	210PB
FL4.5	23.3	2.59	4.5	bulk sediment	210PB
FL.5.0	30.1	2.97	5	bulk sediment	210PB
FL5.5	35.7	3.38	5.5	bulk sediment	210PB
FL6.0	42.3	3.97	6	bulk sediment	210PB
FL6.5	51.6	4.65	6.5	bulk sediment	210PB
FL7.0	59.6	5.47	7	bulk sediment	210PB
FL7.5	67.7	6.44	7.5	bulk sediment	210PB
FL8.0	74.8	7.80	8	bulk sediment	210PB
FL8.5	90.7	11.26	8.5	bulk sediment	210PB
FL9.0	104.3	16.31	9	bulk sediment	210PB
FL25.2	305	33.00	21.67	Aquatic Grass	AMS14C
Fl40.3	669	38.00	36.64	Wood	AMS14C
FL76.5	1320	34.00	63.44	Conifer Cone	AMS14C
FL98.6	1767	35.00	84.62	Wood	AMS14C
FL111.5	1958	35.00	94.34	Wood	AMS14C
FL135.2	2262	36.00	115.31	Wood	AMS14C
FL146.5	2446	36.00	125.41	Wood	AMS14C

### Grain size analysis

**Fig 3:**
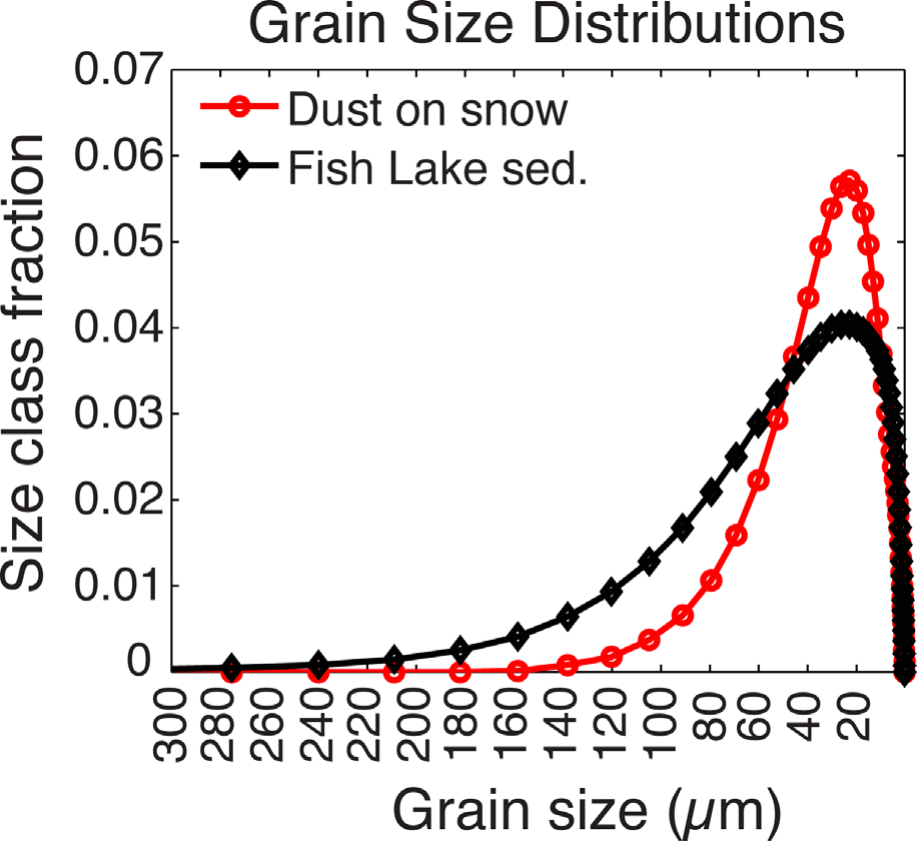
Grain Size. Grain size distributions of dust off snow (red) and Fish Lake sediment (black). The distribution of sediment grain sizes is an average of 337 down-core sediment samples.

**Fig 4:**
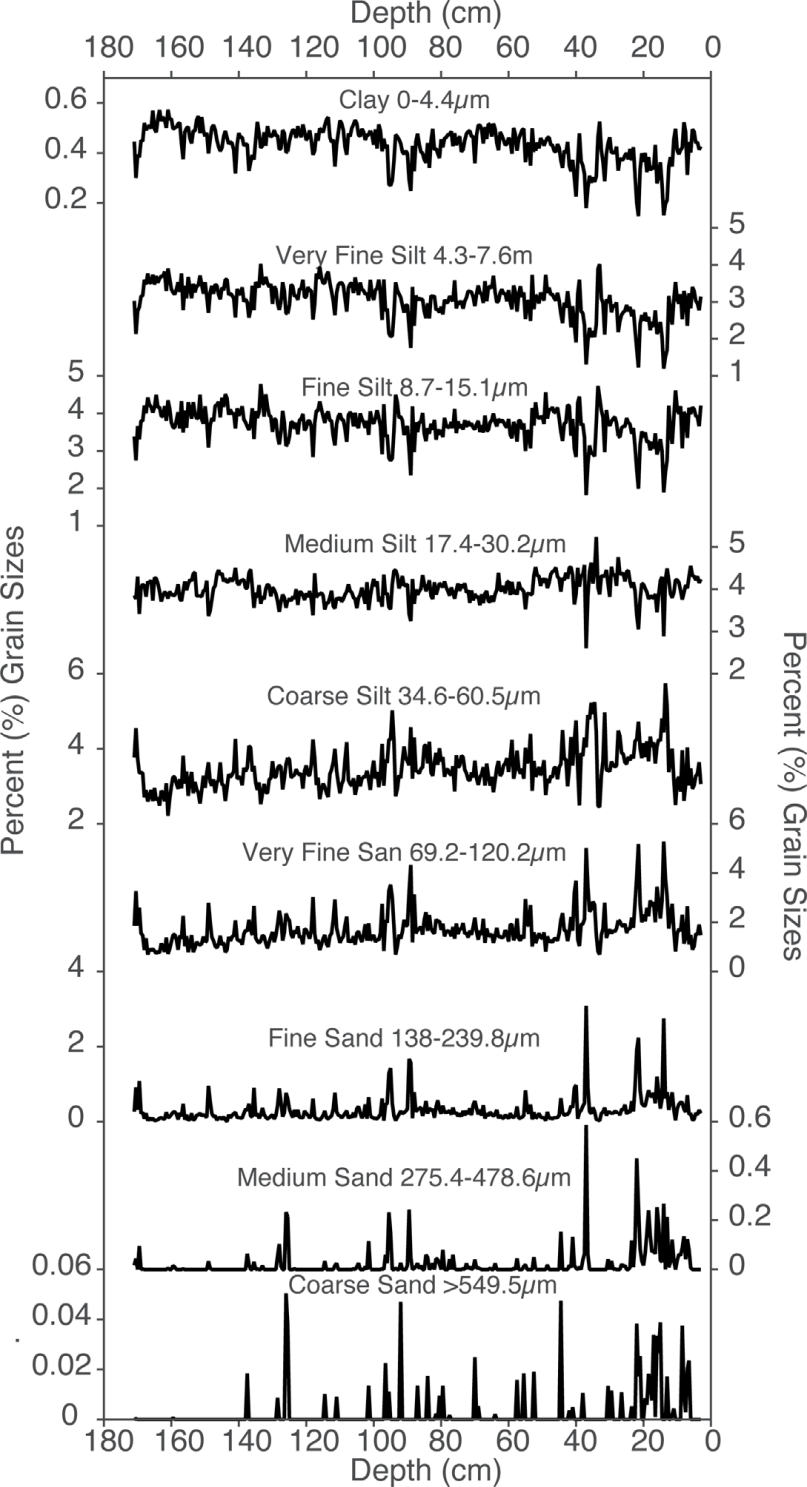
Down core particle size fractions. The dust grain sizes in the sediment core were characterized as those smaller than or equal to 15.1μm (fine silt).

Particle size distributions of lake sediment and local dust on snow suggest that the sediment is composed predominantly of dust grain sizes (Fig 3). Fish Lake sediment contains coarser material than pure dust, indicating inputs from weathering and decomposition of local bedrock. Particle sizes from clay through fine silt (characterized here as particles less than or equal to the 15.1μm) have a coherent down core pattern (Fig 4). Because of this consistent down-core pattern we classified “dust” as particle sizes less than or equal to 15.1μm. Although many dust particles are larger than 15.1μm (e.g. [17] and Fig 3), the consistent down-core pattern of particle sizes less than or equal to 15.1μm (Fig 4) indicate these size classes have the least influence of locally derived material. In contrast, particles sizes larger than 34.6μm (coarse silt and up) also have a consistent pattern, reflecting a more predominant source of local terrigenous input. Medium silt (17.4μm-30.2μm) likely includes large contributions of both dust and local material.

### Geochemical analysis

**Fig 5:**
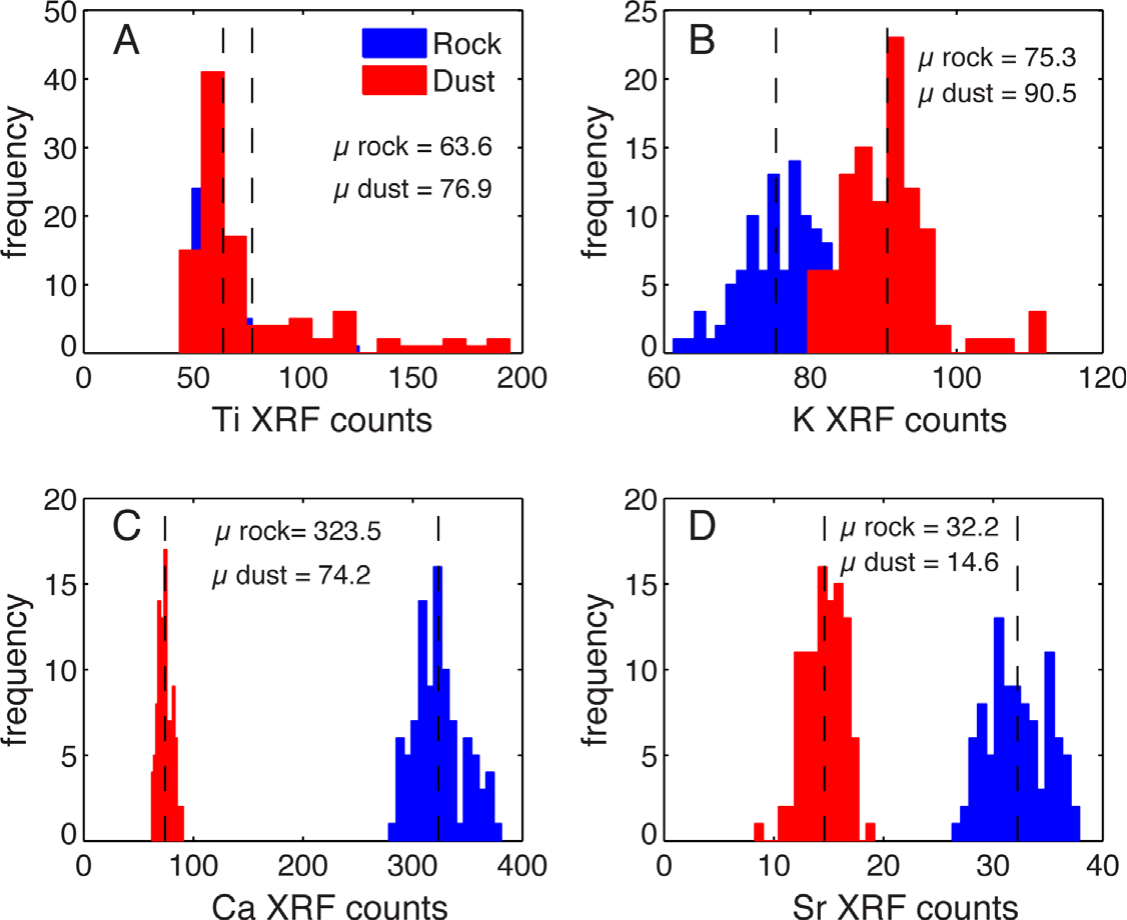
Dust versus bedrock geochemistry. Histograms showing the frequency of elemental abundances characterized using μXRF counts. Counts increase to the right on the x-axis. Note the x-axis scale differs between plots. (A) Titanium has similar abundances in local bedrock and in windblown dust. (B) Potassium has slightly higher abundance in windblown dust. (C) Calcium has moderate to high abundance in windblown dust and much higher abundance in local bedrock. (D) Strontium has slightly higher abundance in local bedrock than windblown dust, but counts were too low to measure in the sediment reliably.

Windblown dust and local bedrock have similar titanium counts; dust is slightly enriched in potassium, whereas calcium and strontium are higher in the local bedrock (Fig 5A–5D). Strontium concentrations however, were too low to measure in the sediment reliably. Calcium shows the greatest difference between local rock and windblown dust (Fig 5C). Calcium is present in moderate to high concentrations in windblown dust collected from southwestern landscapes [5,47]; however, μXRF analysis shows that calcium abundance in the bedrock around Fish Lake is over 4 times higher than in dust deposited on local snow (Fig 5C).

**Fig 6:**
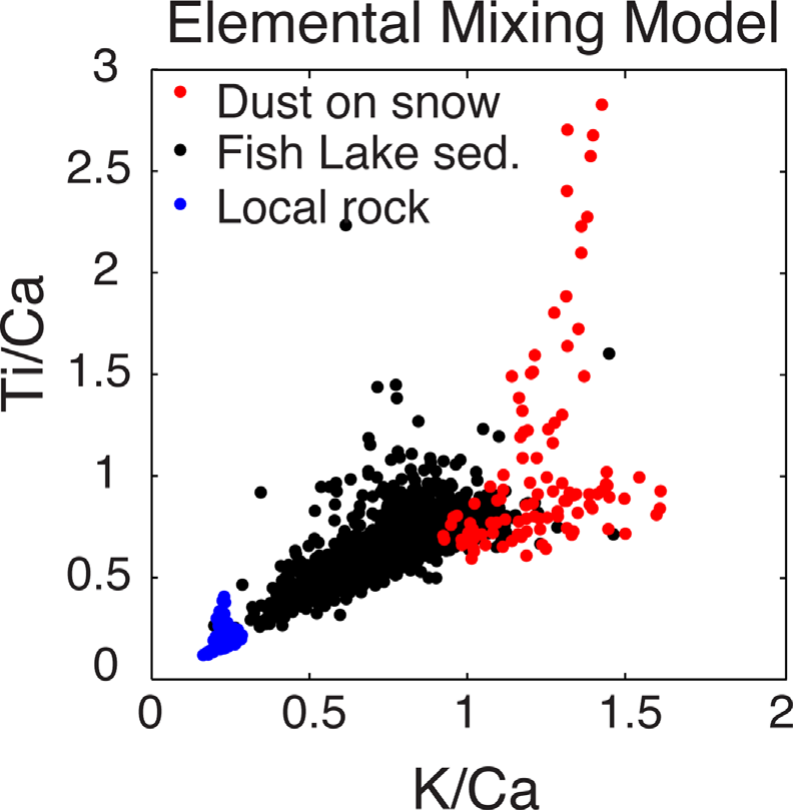
Elemental mixing model. Elemental μXRF ratio/ratio end-member mixing model including titanium, potassium, and calcium, showing sediment (black) distributed between bedrock (blue) and windblown dust (red) end-members. Points in the mixing model represent individual μXRF measurements taken at hundreds of different locations on pulverized dust and bedrock samples and the entire length of the sediment core, measured at 25-micron resolution.

**Fig 7:**
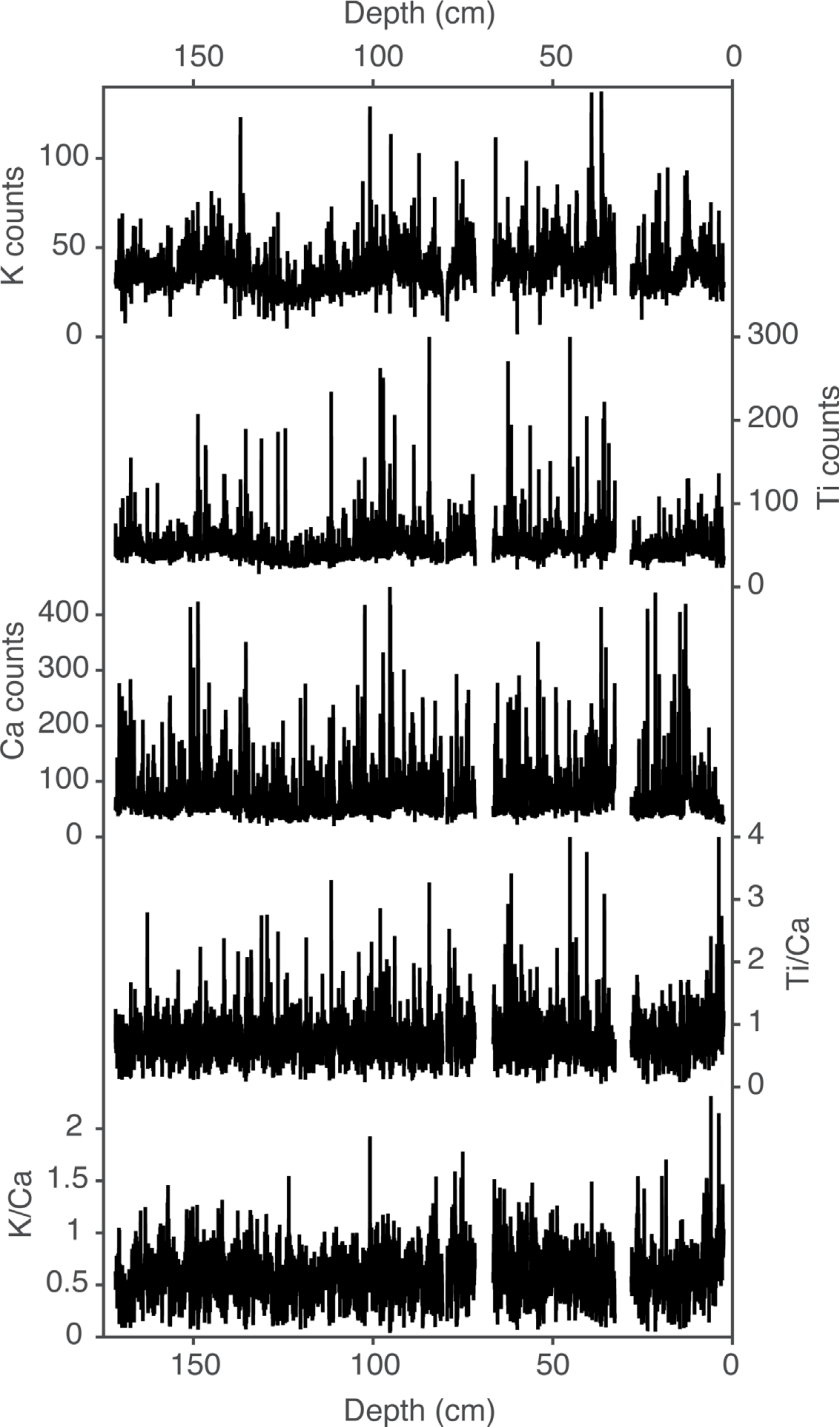
Elemental abundances. Down core μXRF elemental abundances including Ti, K, and Ca counts, and Ti/Ca and K/Ca elemental ratios.

**Fig 8:**
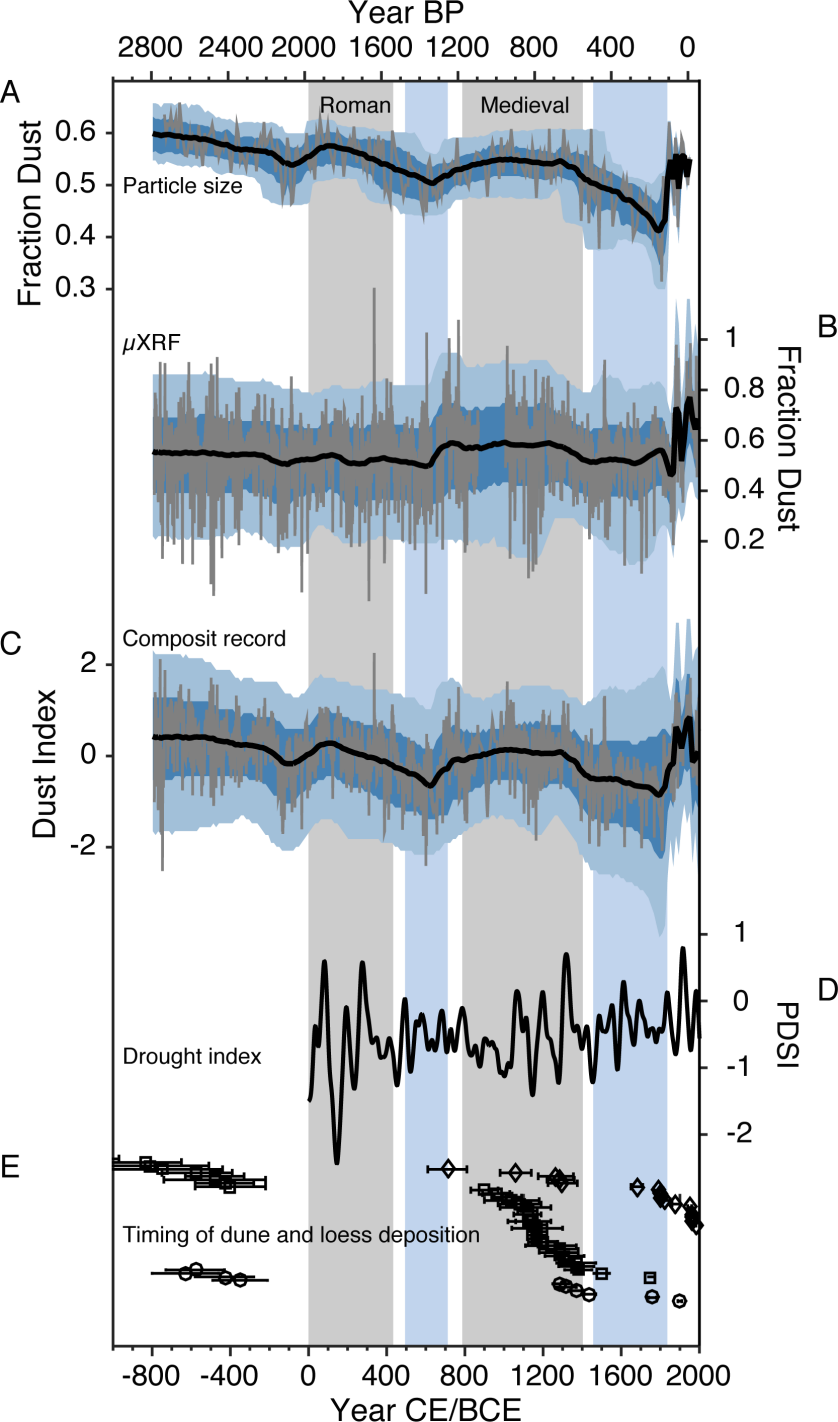
Comparison of Fish Lake dust records with regional drought indicators. (A) Fish Lake grain size dust record with (B), μXRF dust record. (C) Composite Fish Lake dust record (combined particle size and μXRF estimations). Unsmoothed data are shown in grey, and the median age-depth ensemble is shown in black, with dark and light blue bands showing the associated 1 and 2 sigma error bands respectively. (D) Southwestern PDSI [48], smoothed with a 70-year cubic smoothing spline. (E) Dune deposition dates from the Great Sand Dunes National Park (diamonds) [24], and dune (squares) and loess (circles) activity dates from the Great Plains [26]. The y-axis on panel E has no units: the dates are distributed vertically so they don’t obscure one another. Vertical grey bars denote intervals of Roman and medieval aridity. Vertical blue bars show intervals of more stable Southwestern PDSI.

The ratio/ratio scatterplot using Ti/Ca on the y-axis and K/Ca on the x-axis enables the isolation of dust and bedrock contributions to the lake sediment (Fig 6). Local bedrock has low Ti/Ca and K/Ca ratios, and forms a relatively small bedrock end-member. Wind-deposited dust on regional snowpack integrates a variety of geochemical source regions and forms a broad dust end-member. Titanium concentrations are widely variable between dust measurements (e.g. Fig 6A and S5A Fig) which in part contributes to the broad distribution of the dust end-member in Fig 6. Fish Lake sediment, a mixture of the local bedrock and dust, is distributed between the end-members (Fig 6). Down core μXRF estimated elemental abundances including Ti/Ca and K/Ca rations are shown in Fig 7. Because of the wide range of Ti in dust, we used K/Ca ratios in the mixing model to estimate the fraction of dust in Fish Lake sediment back through time (Fig 8B).

### Dust records

On average, Fish Lake sediment contains 54% dust sized particles (sizes smaller than or equal to 15.1μm calculated from the un-interpolated long sediment core). Dust particle size concentrations range from 31.8% to 65.9% (note these are averages of continuously sampled 0.5 cm intervals down core). Preindustrial (periods of the record before 1800 AD) contain 54.1% dust sized particles. After 1800 the sediment core contains an average of 53.0% dust sized particles. There is an overall negative trend in the pre-industrial dust sized particle fraction (Fig 8A). Both the Roman and medieval intervals had increases in the relative amount of dust-sized grains, and the mid-1800s AD saw a reversal in the negative dust grain size trend.

The geochemical record (Shown in Fig 8B) estimates Fish Lake sediment over the past ~3000 years is composed of an average of 55.3% dust, relatively close to the particle size estimation. The high-resolution geochemical record (binned to annual resolution) ranges from -3% to 126.1% dust. Sediment samples with K/Ca ratios that are smaller than the mean of the bedrock end-member or that exceed the mean of the dust end member appear to have less than 0% or more than 100% dust respectively. Preindustrial sediment averages 54.8% dust. Medieval dust percentages (750–1400 AD) are on average 59.0% dust. Post-medieval sediments (1400–1800 AD) contain an average of 53.1% dust. Dust concentrations increase to an average of 61.4% since 1800 AD.

The composite record, built by combining the dust records (4 records where the short cores overlap, and 2 farther back in time), is imperfect but may be a closer approximation to past dust deposition than any one record alone (Fig 8C). The composite record has index (normalized) units, showing relative changes in dust contribution to the sediment record. The overall downward trend from the grain size record is in part retained in the composite record, as are increases in Roman period dustiness. Medieval dust increases in the composite reflect the combined increases in the μXRF and grain size records. The composite record also shows the pronounced increases in dust concentrations beginning in the mid 1800s AD and has an associated decrease in the error at this point.

## Discussion

### Sources of uncertainty

The grain size and μXRF dust estimation methods have their own strengths and weaknesses. Grain size has been used in a wide variety of settings to characterize eolian contribution to lake sediments (e.g. [14,39–44]). Nonetheless, particle sizes alone cannot distinguish between windblown dust and locally derived grains of the same size class. Most lakes accumulate silt and clay sized particle in their depocenter, and some portion of the Fish Lake “dust” grain size fraction record is locally derived. We have attempted to minimize the impact of local sediments on the grain size record by choosing a lake with a relatively small catchment area, yet we are unable to fully quantify the influence of locally sourced dust sized particles on the grain size record. Because of this source of uncertainty, relative changes in dust grain-size abundance are more meaningful than absolute values for addressing the research question regarding the presence and timing of past dust variability. It is also worth noting that the Fish Lake water catchment is in an area that has been untouched by substantial human influence, and so the only explanation for the dramatic post-1885 increase in dust-sized sediment is that it reflective of the large increase in dust-loading seen in our XRF record, but also in other studies (e.g. [17,22]). This gives us further confidence that our grain-size dust record is indeed useful as a dust proxy record.

In contrast, grain size is not susceptible to changes in dust sources and associated changes geochemistry. While the Colorado Plateau is the dominant dust source region to the San Juan Mountains, individual dust storm sources vary widely [3]. We have in part accounted for changes in source geochemistry in the μXRF record by integrating up to eight dust events that occurred before we collected samples in April 2012 [31]. The variability of dust source geochemistry was reflected by the relatively large dust mixing model end-member (Fig 7).

Rocky mountain soils are enriched in aeolian dust [49]. Re-worked dust from soil deposits would not be contemporaneous with the atmospheric deposition and could confound the dust records to some extent. Again, the Fish Lake catchment area is small (0.7 km^2^), and only one small stream enters the lake from the Southeast, minimizing the potential for re-worked dust deposits to confound the Fish Lake record.

Using calcium in the geochemical mixing model may be another source of uncertainty. Sedimentary dust source regions are high in carbonates, and wind-deposited carbonates have a propensity for dissolution [50]. Fish Lake sediments contain no carbonates indicating the carbonates have been removed through dissolution before accumulating in the sediments.

We also tested the reproducibility of the methods on short and long cores. The μXRF record was well reproduced (S7A Fig). The lower resolution grain size record had poorer reproducibility (S7B Fig). There is a greater average dust grain size fraction in the short core, potentially related to different sedimentology at the short-core site. Nonetheless, long-term variability is relatively consistent between the short and long grain size records, both showing recent dust increases.

Finally, an important caveat is that both dust records characterize the fraction of dust in the lake sediment rather than true dust flux. To estimate accurate fluxes, we would need a more precise age model (such as varved sediment) and precise density measurements at the sampling resolution. Both a change in dust or a change in local sediment input would influence the sediment dust fraction.

### Links to Southwestern aridity

The Fish Lake dust records (Fig 8A–8C) provide new insights on Southwestern dustiness and aridity. In spite of the potential sources of uncertainty discussed above, these new records are largely consistent with previous San Juan Mountain dust reconstructions during the last 100 years [17] (S7 Fig), lending confidence to our interpretation of the records. Furthermore, shifts in past dustiness may in part correspond with shifts in Southwestern drought variability as characterized by tree-ring reconstructed Southwestern Palmer Drought Severity Index (PDSI; Fig 8D; 20 grid points averaged, 32°N to 40°N and 105°W to 115°W, and smoothed with a 70 year cubic smoothing spline; [48]). S1 Table shows the significance of mean-state shifts in the dust and drought records (periods delineated by the vertical bars in Fig 8). Low dust deposition occurs notably during the post-medieval (~1450–1850 AD) period, when smoothed PDSI indicates the Southwest experienced less persistent, and less severe, droughts on average. Low dust deposition in the post-medieval period is interrupted by one of the highest dust peaks between 1540 and 1555 AD in the in both short and long μXRF records (Fig 8B and S7A Fig). Although not captured by the lower resolution grain size record, this dust peak is within radiocarbon age error of a well-known multidecadal 16^th^ century Southwestern megadrought [51], and could reflect associated dustiness. The Fish Lake records also indicate a medieval period of relatively high dustiness occurred between ~750 AD and 1400 AD. High medieval dust levels are coincident with widespread increases in drought area [52]. Before the medieval period, the dust records show a moderate decrease in dust deposition between 500 and 700 AD. This decrease in dust deposition corresponds with more stable PDSI (fewer persistent droughts and pluvials), but not significantly wetter conditions. Further back in time, the records show significant dust increases coincident with major oscillations in the PDSI between ~1 AD and 400 AD. The grain size record also shows elevated dustiness between 900 BC and 500 AD, with levels comparable to peak dustiness during the 20^th^ century.

Differences between the dust records and tree-ring reconstructed PDSI could be caused by: 1) the influence of perennial grasses and biologic soil crusts in dust source areas, which have unique responses to drought and are more important for stabilizing soils than trees [53], 2) removal of tree-ring biological growth curves reducing the low frequency component of tree-ring records [54], and 3) the inherent uncertainty of radiocarbon age control, which is decadal scale at best before the more accurate ^210^Pb chronology begins in the 1800s. In any case, the dust records provide an independent, long-term perspective on aridity and soil stabilization.

The Fish Lake records also confirm earlier work highlighting the anomalous dustiness related to 19^th^ century mass livestock introductions and human land use [17]. Livestock were initially introduced in low numbers into the Southwest as early as the mid 1500s with the first Spanish explorers [55]. Completion of the railroad in the mid to late 1800s enabled an exponential increase in livestock populations, with numbers of sheep and cattle in the millions. Fragile desert ecosystems were quickly denuded of grasses and vegetation, resulting in widespread arroyo cutting, soil destabilization, and landscape changes across the Southwest [56]. By the 1920s livestock numbers had stabilized and begun to decline. Livestock declines however, came shortly before the 1930s dust bowl drought, followed by the 1950s drought. Data from μXRF suggests there were two periods of anomalous dustiness related to human land use not captured by the lower resolution grain size record. Dustiness as recorded by μXRF first peaked between 1885 and 1905. A drop in dustiness occurred between 1910 and 1925, coincident with a decadal length Southwestern pluvial [57]. The μXRF record shows dust began increasing again by the 1930s, rising through the 1950s when dust stabilized and then declined. Declines in dust loading were probably related to a decrease in livestock abundance coupled with land management practices and the end of 1930s and 1950s droughts. The μXRF record suggests that dust levels increased again somewhat since the mid 1980s perhaps associated with changes in land use and or recent droughts in the Southwest. Overall, the μXRF record indicates that recent dustiness is more anomalous than the grain size record, and is consistent with previous reconstructions (S8 Fig) [17].

The chronology of dune and loess activity from around the western and central US provide an independent, yet lower resolution line of evidence that widespread dusty conditions occurred during and before major human land use (Fig 8E). The Great Sand Dunes National Park, located 115 km northeast of Fish Lake, experienced medieval and recent dune activity consistent with Fish Lake [24]. Evidence from the Great Plains shows some recent dune and loess activity during the last 150 years [25–26]. Dune mobilization and loess deposition also occurred in the Great Plains during medieval times and before 300 BC (Fig 8E) [26]. Coincident dust increases at Fish Lake, especially evident during the medieval period (shown in both grain size and μXRF records) and before 300 BC in the grain size record (Fig 8A), may reflect widespread impacts of past aridity.

## Conclusions and Implications

Dust flux has varied considerably in the Southwest over the past several millennia, implying that Southwest landscapes undisturbed by humans and their livestock can nonetheless become significant dust sources. Recent dust levels are anomalous, but these new records indicate persistent dustiness has also occurred in the past. Consistent with previous research in arid regions, our findings indicate that regional dustiness is closely linked with regional aridity [58]. The medieval period was especially dusty, coincident with increased drought persistence and area as recorded by tree-ring reconstructed drought records [48,52]. Dune and loess deposits in the Southwest and the Great Plains indicate that some dusty periods at Fish Lake were likely related to widespread aridity. Recent research has documented impacts of dust on snow causing reductions in runoff and streamflow (e.g. in the Colorado River; [18–20]). Furthermore, mineral dust aerosols have also been implicated in past precipitation suppression [59]. It is not yet clear if preindustrial dust levels at Fish Lake were sufficient to suppress precipitation, but evidence suggests that atmospheric dust loading amplifies the impacts of drought [60]. As the Earth warms, the Southwest is projected to see continued warming, a decrease in mean precipitation, a reduction in soil moisture, and an increase in consecutive dry days [61]. These changes will lead to an increased risk of prolonged drought [62], worsened by warming and increased atmospheric moisture demand [63]. Resulting aridity- and human-driven atmospheric mineral dust loading will amplify severe climate change impacts on water resources [18,20] and human health [64–65], exacerbating the regional impacts of anthropogenic climate change.

## Supporting Information

S1 Dataset **Supplemental Dataset:** The Supplemental dataset contains the data used in this publication including (1) the composite dust record (plotted in Fig 8C), (2) μXRF Ti, Ca, and K down-core data on short and long cores, (3) μXRF dust fraction record (plotted in Fig 8B), (4) Grain size fraction ≤15.1μm (Plotted in Fig 8A), (5) raw long-core grain size data, (6) raw short core grain size data, (7) Wolf Creek Pass dust grain size data, (8) Wolf Creek Pass dust μXRF data, (9) Fish Lake rock μXRF data, and (10) Fish Lake turbidite intervals (still included in the XRF down core Ti, Ca, and K measurements and grain size data). The XRF data here have been linearly adjusted for the shrinkage during epoxy resin imbedding.
(XLSX)

S1 Fig Sampling Fish Lake, July 26, 2009. (TIF)

S2 Fig Turbidite deposits. Underwater landslides or large influxes of material washing into the lake deposited as instantaneous packages of sediment formed these features. They are characterized in the sediment core by discrete lower boundaries, with coarse material fining upwards as the flood or landslide deposit settled to the lake floor.
(TIF)

S3 Fig Fish Lake ^210^Pb age model. Dates shown in the filled squares plotted with associated 1σ error.
(TIF)

S4 Fig Calcium versus coarse-grained sediment. Coarse grain sizes in the sediment core compared with μXRF calcium counts showing coarse sections of the core are enriched in calcium concentrations. Increased calcium abundance with coarser grains is consistent with local material being enriched in calcium with respect to wind-deposited dust. Both records are shown here before the turbidites were removed.
(TIF)

S5 Fig Epoxy elemental abundance adjustment. Elemental ratio scatter plots of μXRF counts of dust off snow (red), bedrock from around Fish Lake (blue), and Fish Lake sediment (black). Panels (A-B) show raw μXRF scatter plot Ti versus Ca and K versus Ca counts respectively. Counts are lower in the sediment with respect to the dust and bedrock due to epoxy resin and organic matter filling in between mineral grains. Panels (C-D) show the adjusted fish lake sediment with respect to dust and local bedrock (Shifting sediment elemental counts higher to account for the organic mater and resin induced count reductions), illustrating how the sediment is a mixture of the two sources.
(TIF)

S6 Fig Comparison of grain size and μXRF dust records before turbidities were removed. The μXRF record has been smoothed with a 25 point moving average to reduce noise, and both records have been normalized by their mean and variance.
(TIF)

S7 Fig Record reproducibility. Comparing the reproducibility of grain size and geochemical dust records using short and long cores. Dust μXRF records shown at annual resolution (top) and dust grain size records (bottom) and from short (black) and long (red) cores. The gaps in the μXRF record are sections of sediment that did not imbed properly. The higher resolution μXRF record is more reproducible both in variability and magnitude of dust fraction estimates than the grain size method.
(TIF)

S8 Fig Comparison with previous work. Fish Lake dust records compared with published dust records from the central San Juan Mountains [17]. Senator Beck and Porphery Lakes clearly show recent increases in human induced dustiness. The Fish Lake records confirm the anomalous recent dustiness. Fish Lake also provides a higher resolution perspective on past dust variability, where flux rate estimations from Senator Beck and Porphery Lakes are limited by available age control and sample resolution.
(TIF)

S1 Table Testing for significant differences in records between intervals. Significance level of mean-differences between respective periods in the dust and drought records highlighted by the vertical bars in Fig 8.
(DOCX)
