# Supplementary figures and images for: Three Millennia of Southwestern North American Dustiness and Future Implications
Source: PLoS One. 2016 Feb 17;11(2):e0149573. doi: 10.1371/journal.pone.0149573 (PMC4757576; doi:10.1371/journal.pone.0149573)

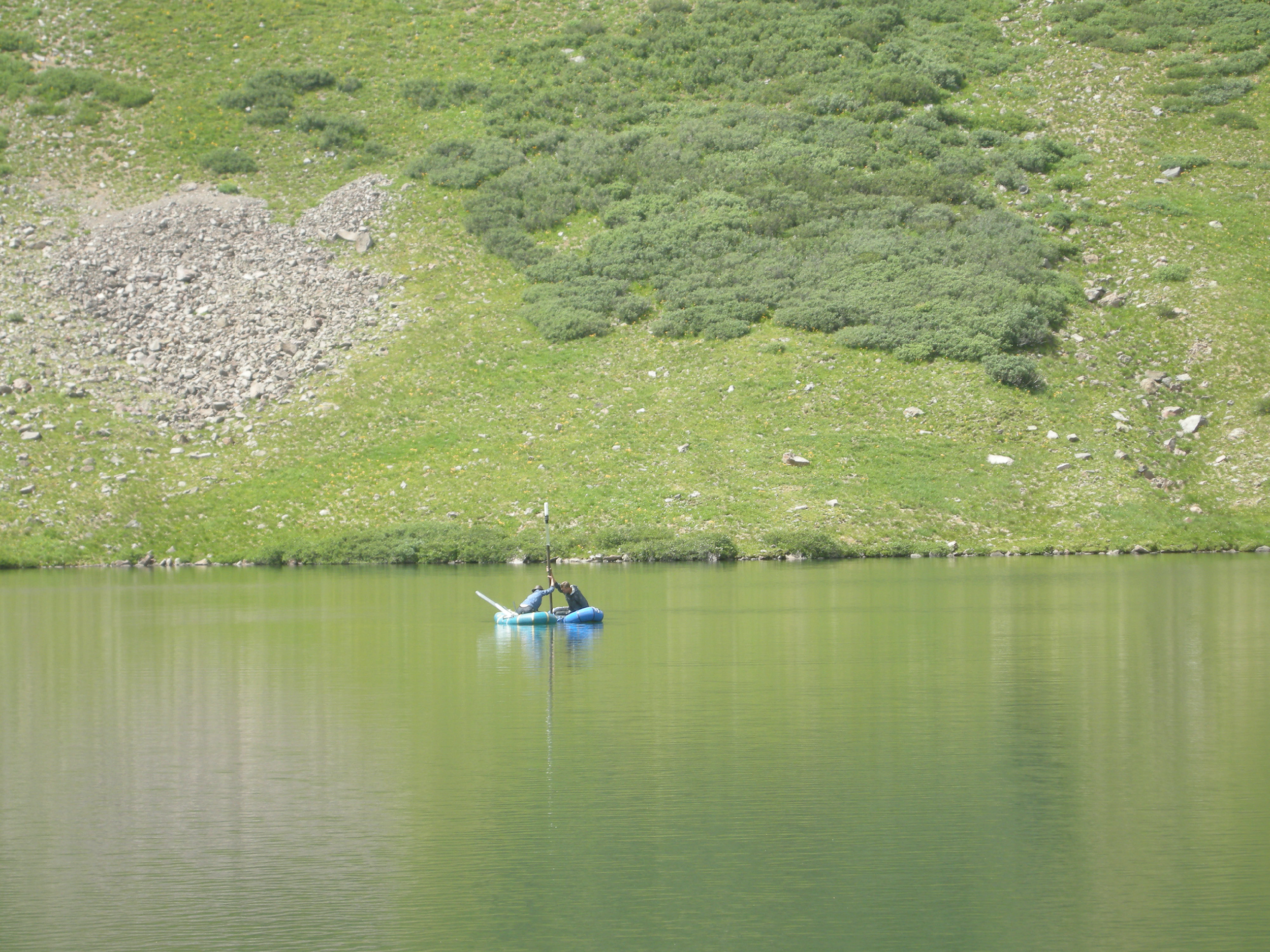

Supplement: S1 Fig — (TIF) [file pone.0149573.s002.tif]

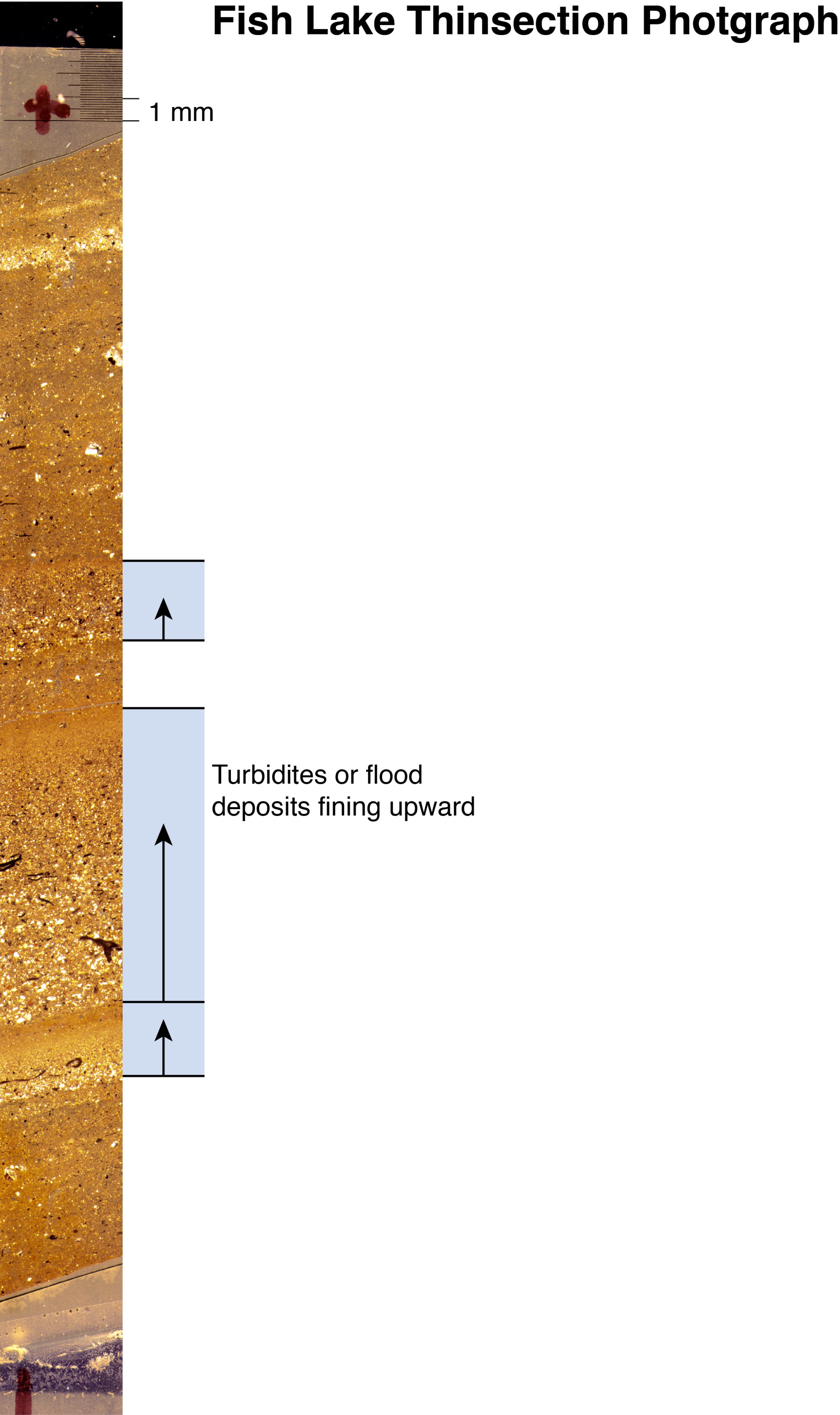

Supplement: S2 Fig — Underwater landslides or large influxes of material washing into the lake deposited as instantaneous packages of sediment formed these features. They are characterized in the sediment core by discrete lower boundaries, with coarse material fining upwards as the flood or landslide deposit settled to the lake floor. (TIF) [file pone.0149573.s003.tif]

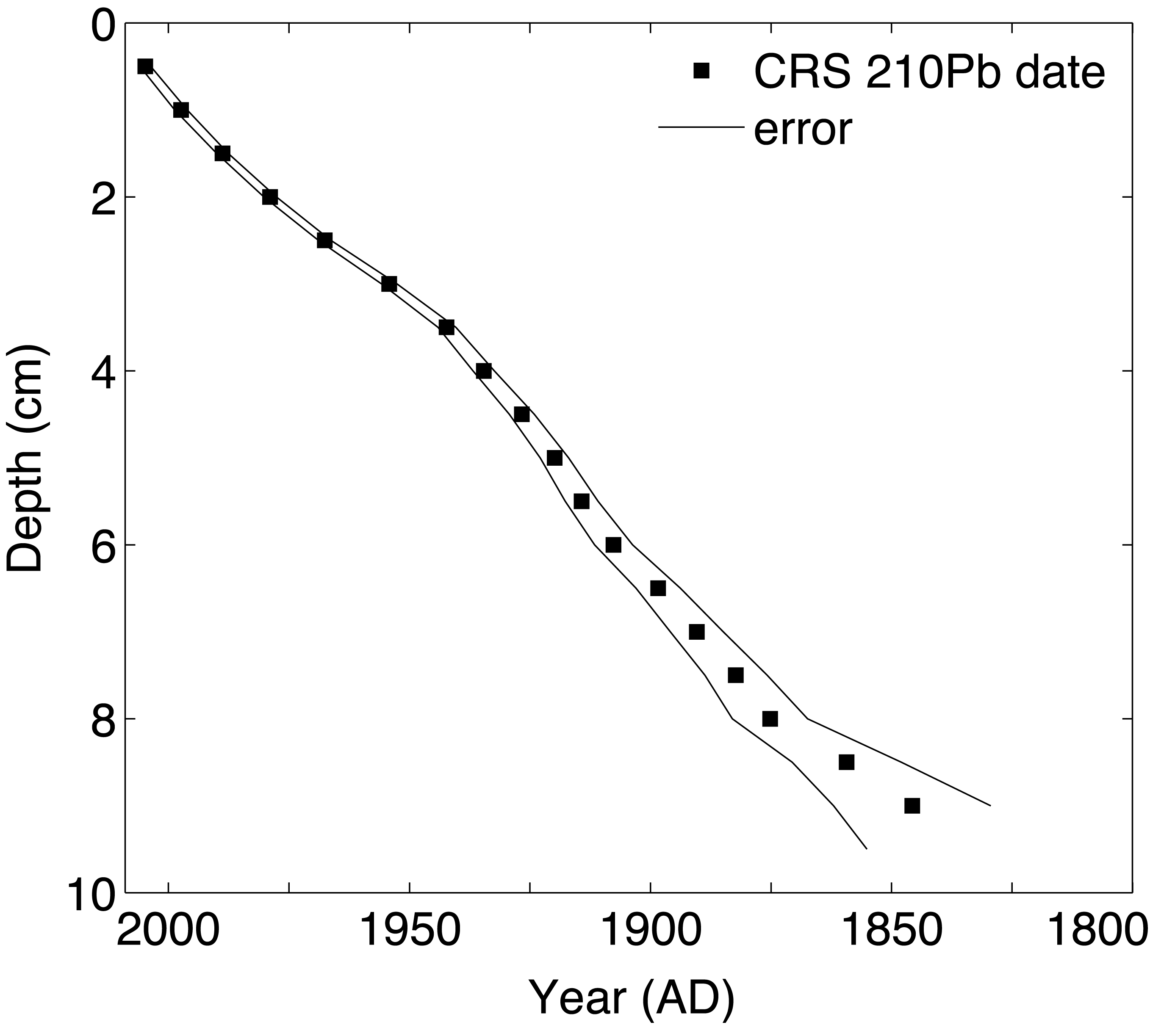

Supplement: S3 Fig — Dates shown in the filled squares plotted with associated 1σ error. (TIF) [file pone.0149573.s004.tif]

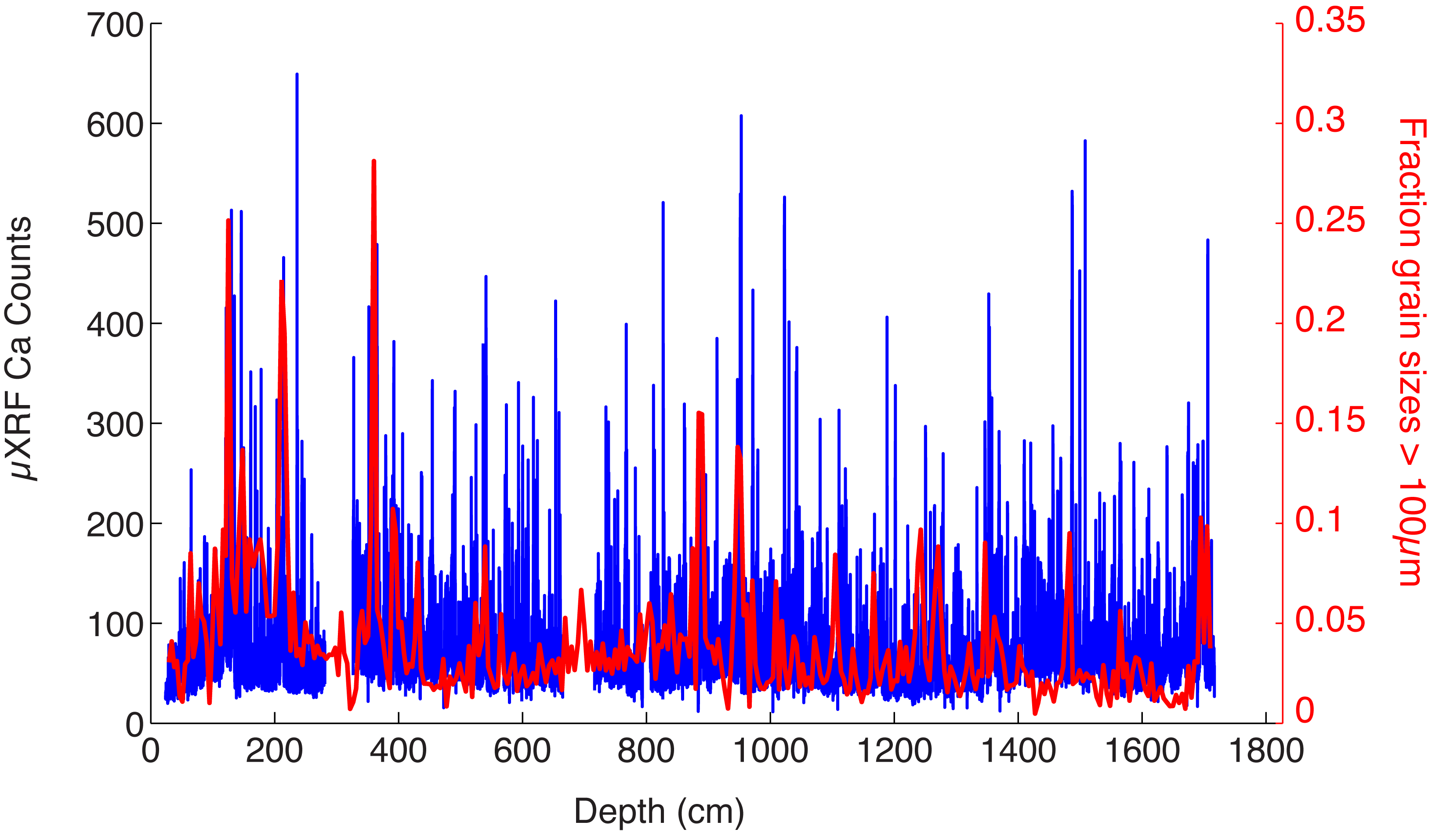

Supplement: S4 Fig — Coarse grain sizes in the sediment core compared with μXRF calcium counts showing coarse sections of the core are enriched in calcium concentrations. Increased calcium abundance with coarser grains is consistent with local material being enriched in calcium with respect to wind-deposited dust. Both records are shown here before the turbidites were removed. (TIF) [file pone.0149573.s005.tif]

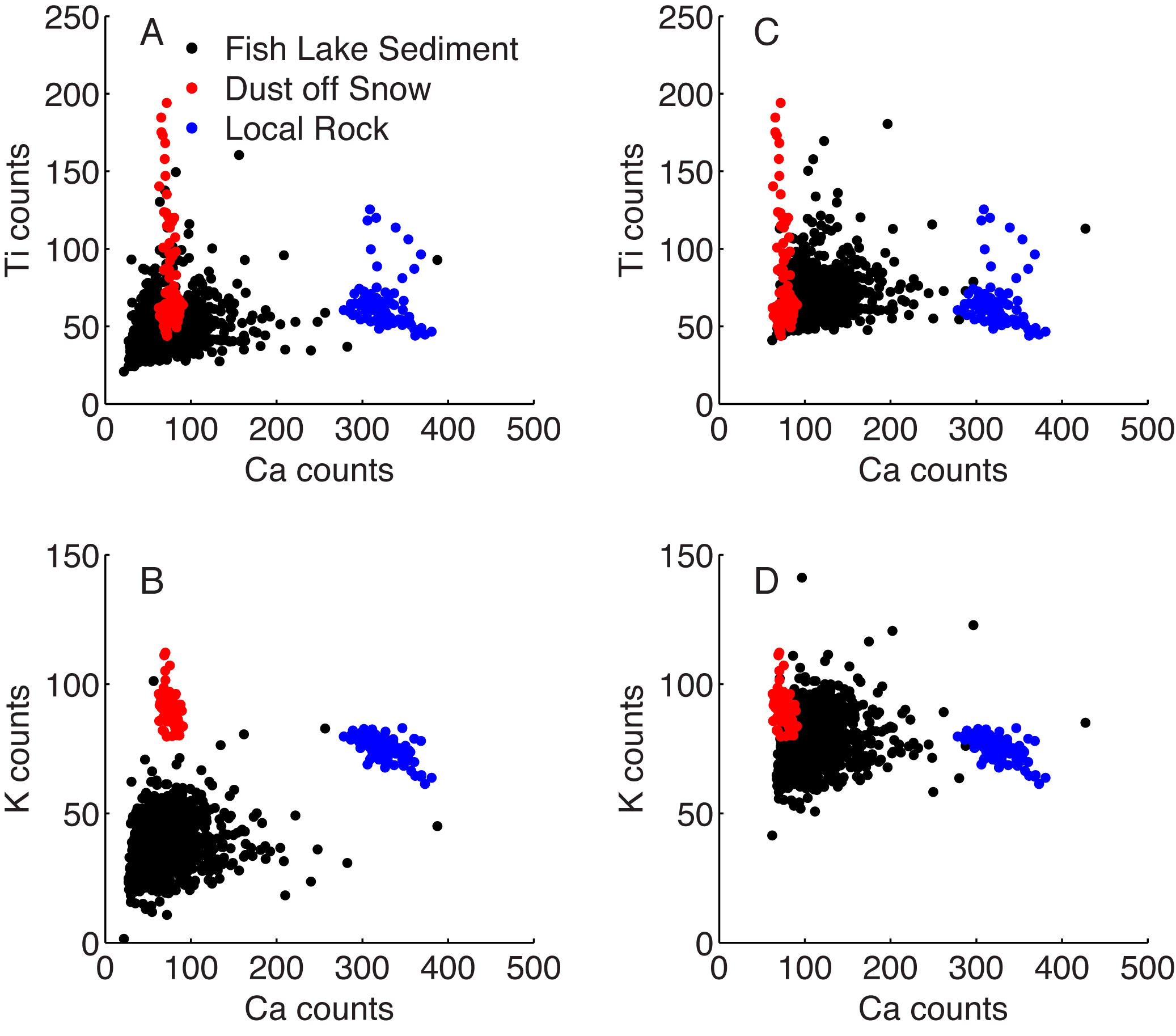

Supplement: S5 Fig — Elemental ratio scatter plots of μXRF counts of dust off snow (red), bedrock from around Fish Lake (blue), and Fish Lake sediment (black). Panels (A-B) show raw μXRF scatter plot Ti versus Ca and K versus Ca counts respectively. Counts are lower in the sediment with respect to the dust and bedrock due to epoxy resin and organic matter filling in between mineral grains. Panels (C-D) show the adjusted fish lake sediment with respect to dust and local bedrock (Shifting sediment elemental counts higher to account for the organic mater and resin induced count reductions), illustrating how the sediment is a mixture of the two sources. (TIF) [file pone.0149573.s006.tif]

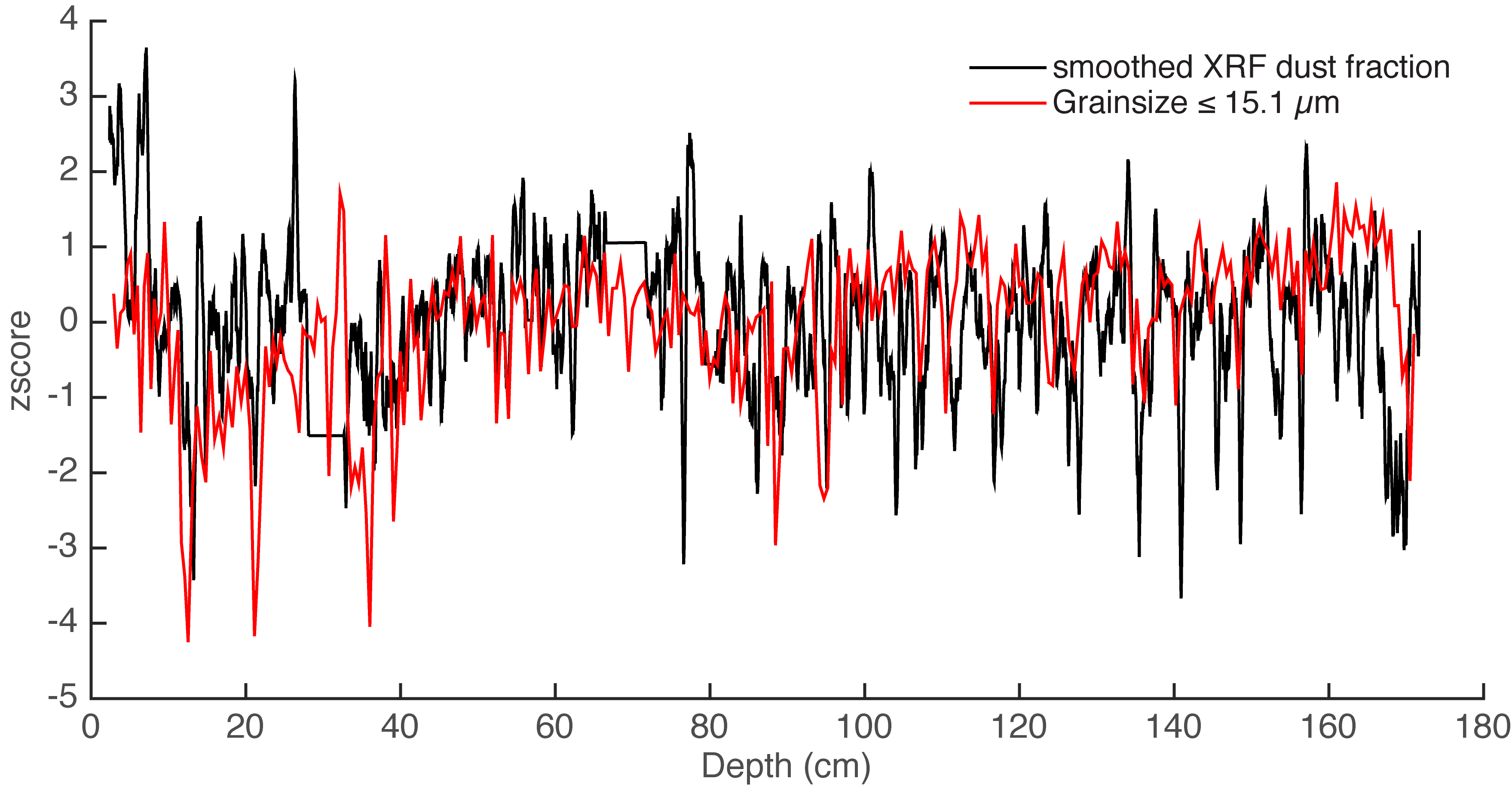

Supplement: S6 Fig — The μXRF record has been smoothed with a 25 point moving average to reduce noise, and both records have been normalized by their mean and variance. (TIF) [file pone.0149573.s007.tif]

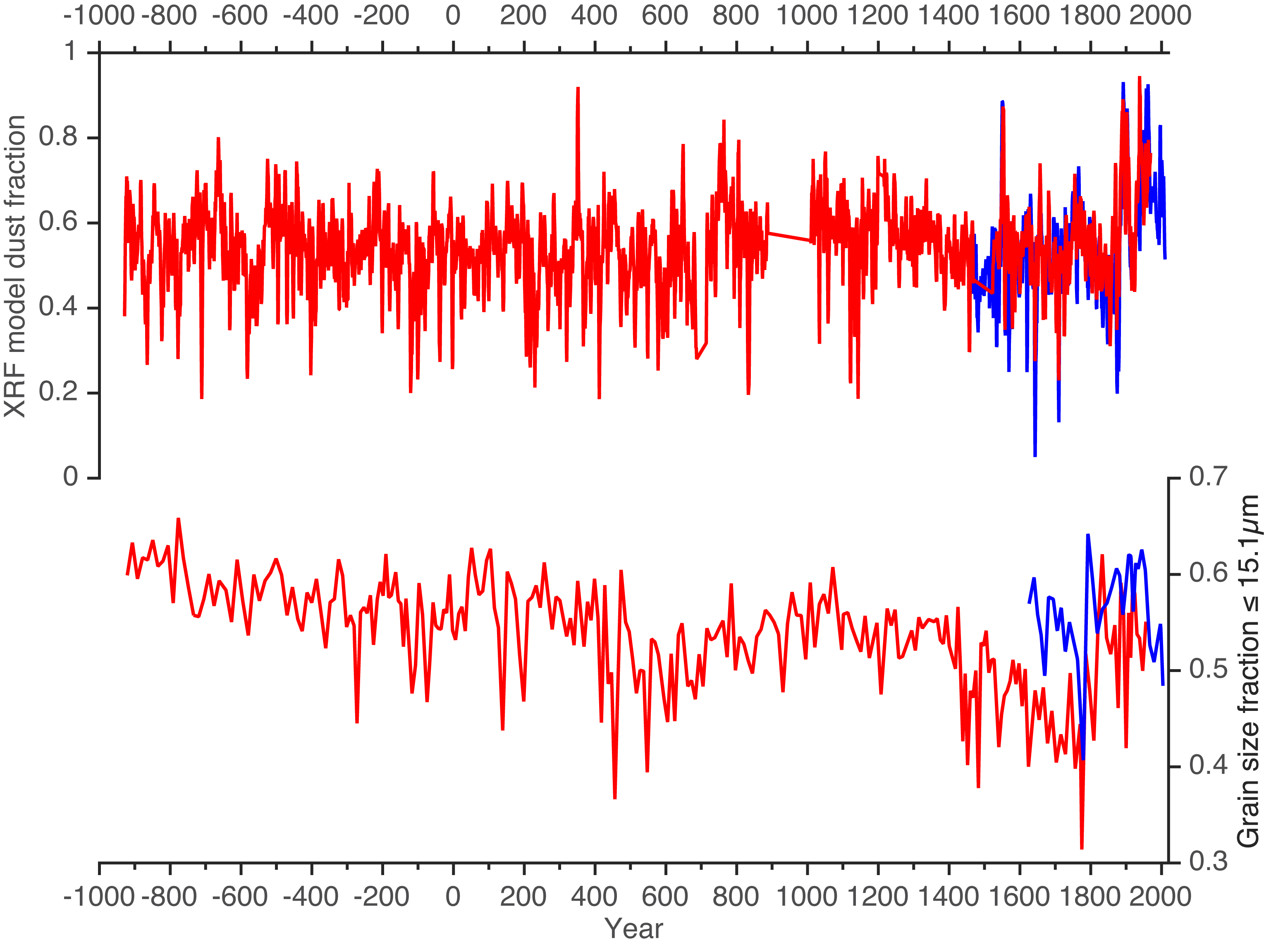

Supplement: S7 Fig — Comparing the reproducibility of grain size and geochemical dust records using short and long cores. Dust μXRF records shown at annual resolution (top) and dust grain size records (bottom) and from short (black) and long (red) cores. The gaps in the μXRF record are sections of sediment that did not imbed properly. The higher resolution μXRF record is more reproducible both in variability and magnitude of dust fraction estimates than the grain size method. (TIF) [file pone.0149573.s008.tif]

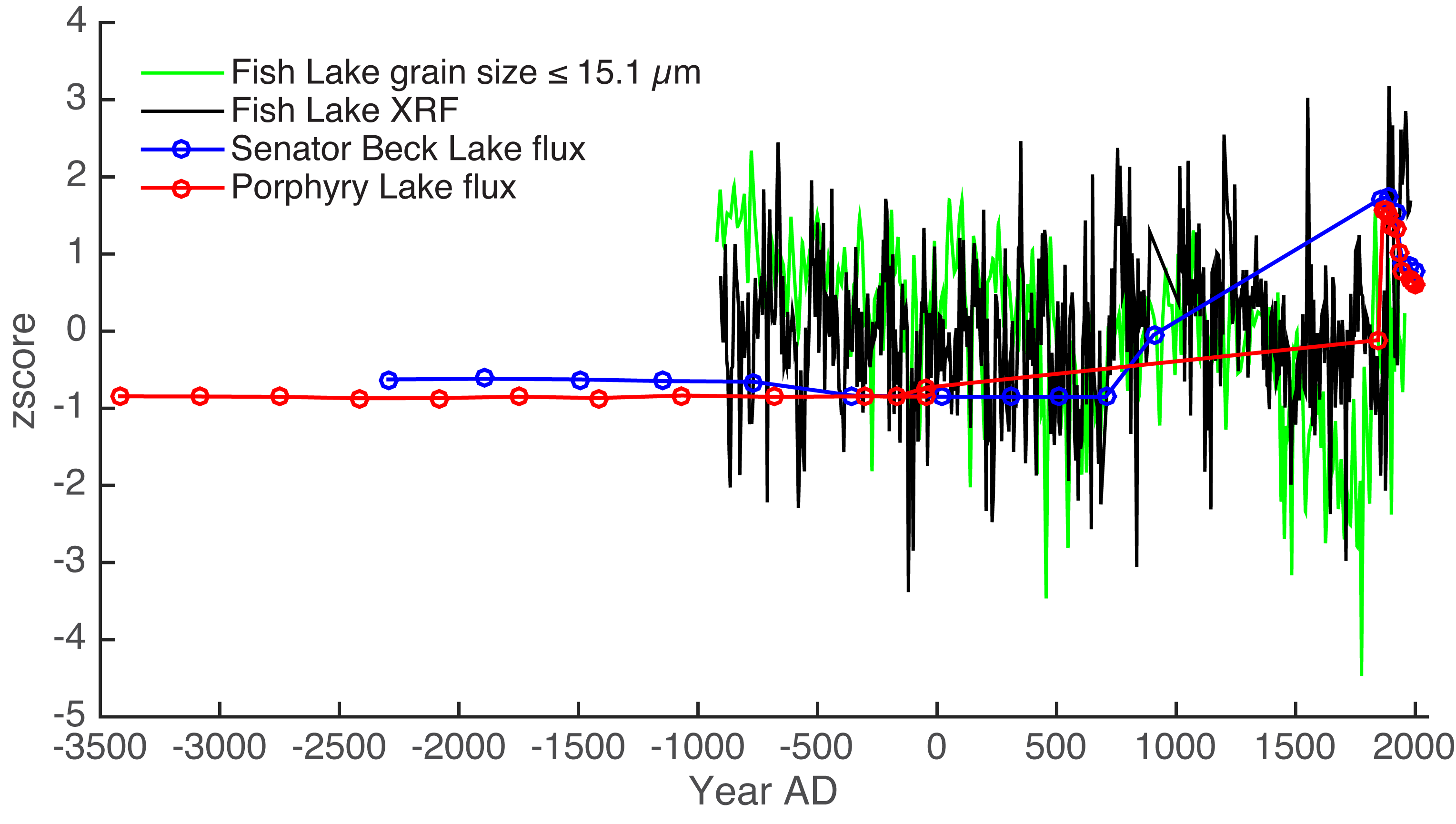

Supplement: S8 Fig — Fish Lake dust records compared with published dust records from the central San Juan Mountains [17]. Senator Beck and Porphery Lakes clearly show recent increases in human induced dustiness. The Fish Lake records confirm the anomalous recent dustiness. Fish Lake also provides a higher resolution perspective on past dust variability, where flux rate estimations from Senator Beck and Porphery Lakes are limited by available age control and sample resolution. (TIF) [file pone.0149573.s009.tif]
