# Supplementary material for: Three Millennia of Southwestern North American Dustiness and Future Implications
Source: PLoS One. 2016 Feb 17;11(2):e0149573. doi: 10.1371/journal.pone.0149573 (PMC4757576; doi:10.1371/journal.pone.0149573)
Supplement: S1 Table — Significance level of mean-differences between respective periods in the dust and drought records highlighted by the vertical bars in Fig 8. (DOCX) [file pone.0149573.s010.docx]

| **Grain size record ≤ 15.1µm** | | |
| --- | --- | --- |
| **Intervals** | 1AD-450AD | 750-1400AD |
| 500AD-700AD | p < 0.01 | p < 0.01 |
| 1450-1850AD | p < 0.01 | p < 0.01 |
|  |  |  |
| **µXRF dust fraction record** | | |
| **Intervals** | 1AD-450AD | 750-1400AD |
| 500AD-685AD | p = 0.06 | p < 0.01 |
| 1450-1850AD | p = 0.60 | p < 0.01 |
|  |  |  |
| **Composite record** | | |
| **Intervals** | 1AD-450AD | 750-1400AD |
| 500AD-700AD | p < 0.01 | p < 0.01 |
| 1450-1850AD | p < 0.01 | p < 0.01 |
|  |  |  |
| **Southwestern PDSI reconstruction** | | |
| **Intervals** | 1AD-450AD | 750-1400AD |
| 500AD-700AD | p < 0.01 | p = 0.27 |
| 1450-1850AD | p < 0.01 | p < 0.01 |

**S1 Table.** **Testing for significant differences in records between intervals.** Significance level of mean-differences between respective periods in the dust and drought records highlighted by the vertical bars in Fig 8.
